# Enhancing Cardiovascular Autonomic Regulation in Parkinson’s Disease Through Non-Invasive Interventions

**DOI:** 10.3390/life15081244

**Published:** 2025-08-05

**Authors:** Aastha Suthar, Ajmal Zemmar, Andrei Krassioukov, Alexander Ovechkin

**Affiliations:** 1 Department of Neurological Surgery, University of Louisville, Louisville, KY 40202, USA; aastha.suthar@louisville.edu; 2 Kentucky Spinal Cord Injury Research Center, University of Louisville, Louisville, KY 40202, USA; 3 Department of Surgery, United States Department of Veteran Affairs, 800 Zorn Ave, Louisville, KY 40206, USA; ajmal.zemmar@gmail.com; 4 International Collaboration on Repair Discoveries (ICORD), University of British Columbia, Vancouver, BC V5Z 1M9, Canada; andrei.krassioukov@vch.ca; 5 Division of Physical Medicine and Rehabilitation, Faculty of Medicine, University of British Columbia, Vancouver, BC V5Z 2G9, Canada; 6 GF Strong Rehabilitation Centre, Vancouver Coastal Health, Vancouver, BC V5Z 2G9, Canada

**Keywords:** baroreflex sensitivity, Parkinson’s disease, non-invasive rehabilitation

## Abstract

Background: Parkinson’s disease (PD) often involves autonomic dysfunction, most notably impaired baroreflex sensitivity (BRS), which disrupts cardiovascular homeostasis and contributes to orthostatic hypotension (OH). Pharmacological and invasive treatments, including deep brain stimulation, have yielded inconsistent benefits and carry procedural risks, highlighting the need for safer, more accessible alternatives. In this systematic review, we evaluated non-invasive interventions—spanning somatosensory stimulation, exercise modalities, thermal therapies, and positional strategies—aimed at improving cardiovascular autonomic function in PD. Methods: We searched PubMed, Embase, MEDLINE (Ovid), Google Scholar, ScienceDirect, and Web of Science for studies published between January 2014 and December 2024. Eight original studies (*n* = 8) including 205 participants met the inclusion criteria for analyzing cardiac sympathovagal balance. Results: Five studies demonstrated significant post-intervention increases in BRS. Most reported favorable shifts in heart rate variability (HRV) and favorable changes in the low-frequency/high-frequency (LF/HF) ratio. Across modalities, systolic blood pressure (SBP) decreased by an average of 5%, and some interventions produced benefits that persisted up to 24 h. Conclusion: Although sample sizes were small and protocols heterogeneous, the collective findings support the potential of non-invasive neuromodulation to enhance BRS and overall cardiovascular regulation in PD. Future research should focus on standardized, higher-intensity or combined protocols with longer follow-up periods to establish durable, clinically meaningful improvements in autonomic function and quality of life for people living with PD.

## 1. Introduction

Autonomic dysfunction in Parkinson’s disease (PD) can contribute to a more severe phenotype, with accelerated motor decline, increased fall risk, and reduced survival [1]. Among these autonomic abnormalities, impaired baroreflex sensitivity (BRS) is well documented and underlies dysregulated cardiovascular control, exacerbating adverse outcomes [2,3,4]. Degeneration of central and peripheral autonomic pathways in PD leads to inadequate compensation for blood pressure fluctuations, thereby reducing BRS [4,5,6].

Orthostatic hypotension (OH) is one of the most common and debilitating manifestations of autonomic failure in PD, yet it often remains undiagnosed [7,8]. Clinically, OH is defined by a drop in systolic blood pressure (SBP) ≥ 20 mmHg or diastolic blood pressure (DBP) ≥ 10 mmHg upon standing or during head-up tilt at 60° [9]. Such pressure falls provoke dizziness, lightheadedness, and syncope [9], significantly impairing quality of life and elevating the risk of falls, fractures, and related complications in PD patients [10,11,12]. Despite its high prevalence, OH in PD frequently responds poorly to standard pharmacological treatments [13].

Current management of OH and broader autonomic dysfunction in PD remains constrained by a lack of effective non-invasive rehabilitative therapies. These interventions seek to restore endogenous cardiovascular regulatory mechanisms, potentially offering a safer and more consistent benefit than pharmacological or surgical modalities, which carry side effects and display variable efficacy [14,15].

In this review, we examine the pathophysiological basis of BRS impairment in PD and synthesize emerging evidence for non-invasive rehabilitation techniques aimed at enhancing BRS. Our goal is to highlight patient-centered strategies that may improve autonomic control and ultimately reduce the burden of cardiovascular dysautonomia in PD.

## 2. Materials and Methods

### 2.1. Search Strategy

A systematic literature search was performed to identify peer-reviewed studies evaluating non-invasive interventions for cardiovascular autonomic dysfunction in PD. We queried PubMed, Embase, MEDLINE (Ovid), Google Scholar, ScienceDirect, and Web of Science for articles published between January 2014 and December 2024. Institutional and open-access platforms provided full-text retrieval.

In PubMed and MEDLINE, we employed controlled-vocabulary Medical Subject Headings (MeSH) combined with Boolean operators (e.g., “Parkinson Disease”, “Baroreflex”, “Non-invasive”, “Rehabilitation”). In Embase and ScienceDirect, we used equivalent Entree and keyword queries. For Google Scholar and Web of Science, we prioritized free-text searches with phrase matching and citation tracking to ensure comprehensiveness. This dual approach, incorporating both subject headings and free-text terms, maximized sensitivity while minimizing retrieval of irrelevant records.

### 2.2. Search Terms

The literature search employed a meticulously curated set of keywords and MeSH to ensure the retrieval of studies most relevant to the investigation of non-invasive interventions for cardiovascular imbalances in PD. The primary search terms included “Baroreflex”, “Baroreflex Sensitivity”, “Autonomic Regulation”, “Orthostatic Hypotension”, “Blood Pressure”, “Cardiovascular”, and “Parkinson’s Disease”. These terms were carefully chosen to encompass the physiological and clinical dimensions of cardiovascular dysregulation in PD and its potential therapeutic management.

To enhance the precision and comprehensiveness of the search, Boolean operators such as “AND” and “OR” were systematically utilized. For instance, terms like “Baroreflex Sensitivity AND Parkinson’s Disease” or “Autonomic Regulation OR Orthostatic Hypotension” were combined to capture studies addressing overlapping domains. Truncation and wildcards were also employed where appropriate to include variations in terms, such as “cardiovascular *” to account for related phrases like “cardiovascular regulation” or “cardiovascular dysfunction”.

By employing a structured and strategic approach to search term selection, the methodology ensured that the literature search was both exhaustive and focused, capturing a broad range of studies pertinent to the research question. This thorough application of search terms facilitated the identification of key publications that formed the basis for subsequent analysis and synthesis.

### 2.3. Inclusion and Exclusion Criteria

To ensure relevance and methodological rigor, we applied the following criteria:

Inclusion Criteria

- Original, peer-reviewed human studies published between January 2014 and December 2024 to capture the most recent evidence in emerging neuromodulation protocols.
- PD patients undergoing non-invasive interventions targeting cardiovascular autonomic function.
- Quantitative assessment of BRS or related cardiovascular outcomes (e.g., heart rate variability (HRV), blood pressure responses).
- Presence of an active treatment arm (single-arm or controlled design).

Exclusion Criteria

- Animal or in vitro studies.
- Reviews, meta-analyses or conference abstracts without full quantitative data.
- Studies evaluating exclusively invasive or pharmacological therapies.
- Interventions lacking a defined therapeutic component (e.g., observational, or purely diagnostic studies).
- Investigations including mixed patient cohorts with comorbidities other than PD, without separate PD-specific analysis.
- Participant populations outside adult age ranges (i.e., <18 years).

By applying these criteria, we focused our review on high-quality clinical investigations of non-invasive rehabilitative strategies for cardiovascular dysautonomia in PD.

### 2.4. Screening and Selection

All retrieved records were imported into EndNote (version 20.2.1) for reference management and de-duplication. We conducted a two-stage screening process:

1. Title and Abstract Screening: Two reviewers independently assessed titles and abstracts to exclude clearly irrelevant studies (e.g., those not involving PD, non-invasive interventions, or cardiovascular outcomes). Discrepancies were resolved by discussion.
2. Full-Text Review: Full texts of potentially eligible articles were obtained and evaluated against the predefined inclusion and exclusion criteria (Section 2.3). Each study was confirmed to involve adult PD patients, non-invasive therapeutic interventions, and quantitative measures of BRS or related cardiovascular outcomes.

Studies failing to meet any criterion were excluded, with reasons documented. This systematic approach ensured that only relevant, high-quality clinical investigations were included in the final analysis.

### 2.5. Risk of Bias Assessment

Risk of bias in the included studies was evaluated independently by two reviewers, who remained blinded to author and journal information to mitigate detection bias. We examined each study across key domains:

- Selection Bias: Assessed random sequence generation and allocation concealment methods.
- Performance and Detection Bias: Evaluated blinding of participants, personnel, and outcome assessors.
- Attrition Bias: Reviewed completeness of outcome data and the handling of missing data, as reported by studies (e.g., use of intention-to-treat analyses or specific imputation methods).
- Reporting Bias: Checked for selective outcome reporting by comparing published methods with reported results.

Any disagreements in bias ratings were resolved through discussion until consensus was reached. Additionally, systematic de-duplication in EndNote and cross-referencing against study protocols helped minimize reporting bias. This rigorous, blinded assessment ensured that our synthesis relied on studies with transparent and robust methodological quality.

### 2.6. Data Extraction and Synthesis

Two reviewers independently extracted data from each included study using a standardized form. Extracted variables comprised:

- Participant Characteristics: Mean age, gender distribution, sample size, disease duration, and Hoehn and Yahr classification.
- Intervention Details: Type of non-invasive modality, study design (e.g., randomized controlled trial, single arm), intervention frequency and duration, stimulation or exercise parameters, and comparator conditions.
- Outcome Measures: Primary metrics of BRS (e.g., sequence method, transfer function analysis), secondary cardiovascular autonomic indices (e.g., HRV, low-frequency/high-frequency (LF/HF) ratio), and any reported adverse events.

The extracted data were systematically presented in tables and figures. Adherence to PRISMA 2020 guidelines was achieved to extent possible considering the scope and heterogenicity of included studies. We focused on identifying rehabilitative parameters and protocols consistently associated with BRS improvement, regardless of statistical significance, to inform future optimization. Adverse effects, when reported, were summarized to guide safety considerations.

## 3. Results

### 3.1. Articles Retrieved

**Figure 1:**
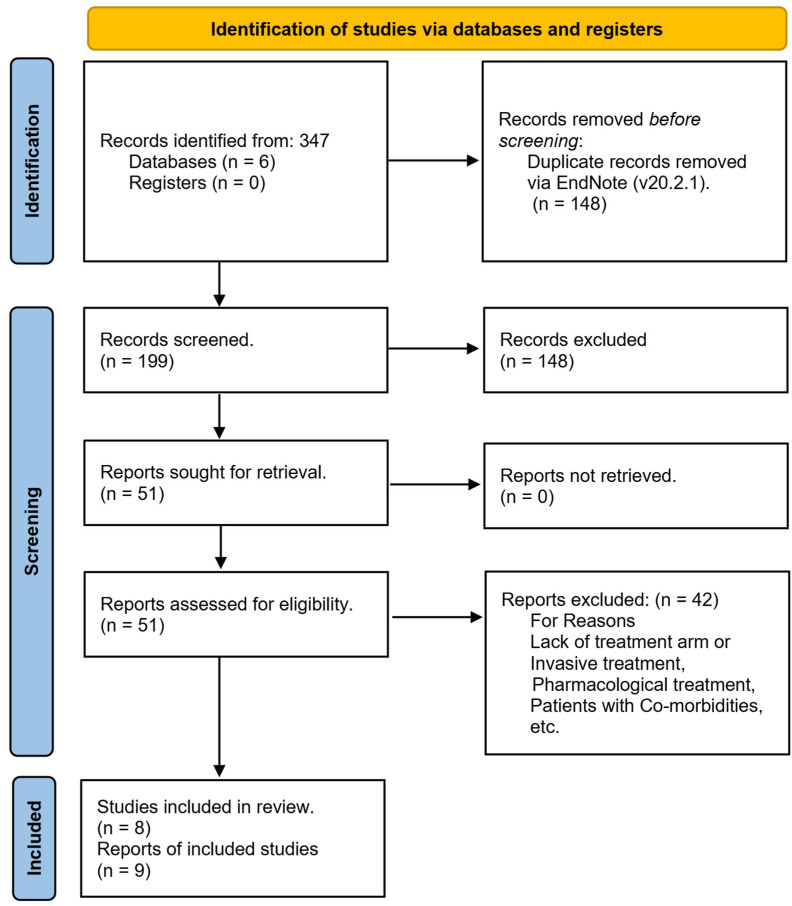
Flow diagram depicting the study selection process.

A systematic literature search yielded 347 records, which were imported into EndNote for the removal of duplicates. Post-deduplication, 199 unique records were subjected to a meticulous screening of titles and abstracts, resulting in the exclusion of 148 records. The remaining 51 full-text articles were then thoroughly assessed for eligibility, leading to the exclusion of 42 articles that did not satisfy the inclusion criteria. Consequently, 9 [16,17,18,19,20,21,22,23,24] reports including 8 original studies and 1 follow up study were selected for data extraction. The study selection process is summarized in Figure 1.

### 3.2. Participants Characteristics

The study population comprised a total of 125 males and 80 females, with one study exclusively involving a single male participant. The overall number of participants was 205, with a distribution between those receiving treatments and those in control or sham groups. The mean age of the participants was 64.32 years, with a disease duration range of 7.17 ± 2.69 years. Notably, all studies included patients who were on levodopa or levodopa equivalent therapy. Characteristics of the study population are summarized in Table 1.

### 3.3. Rehabilitation Characteristics

We identified eight non-invasive interventions that were generally well tolerated by participants: Effective Stimulation (ES), progressive Resistance Training (RT), Whole-Body Cryostimulation (WBC), Partial Weight-Supported Treadmill Training (PWSTT), repeated short-term Dry Immersion (DI), Respiratory Muscle Training (RMT), Head-Up Tilt Sleeping (HUTS), and Automated Mechanical Somatosensory Stimulation (AMSS).

The included studies varied in design, intervention parameters, and duration. Key characteristics are summarized below:

- Effective Stimulation (ES): A randomized clinical trial (RCT) delivered four 6 s sessions of ES (total 2 min) in a single visit [16].
- Resistance Training (RT): In a 12-week RCT, participants completed 24 sessions (twice weekly) of progressive RT [21].
- Automated Mechanical Somatosensory Stimulation (AMSS): An interventional cohort study administered AMSS over 12 days, with two sessions per week (five total sessions) [17].
- Partial Weight-Supported Treadmill Training (PWSTT): A 4-week RCT comprised 16 sessions (four times weekly, 30 min each) [20].
- Dry Immersion (DI): A 4-week controlled clinical trial involved seven 45 min DI sessions (twice weekly) [19].
- Respiratory Muscle Training (RMT): Prospective case–control studies implemented RMT over 12 weeks, with 120 sessions (twice daily, 30 min each) [22,23].
- Whole-Body Cryostimulation (WBC): A one-week pilot study delivered ten 2 min WBC sessions (twice daily) [18].
- Head-Up Tilt Sleeping (HUTS): A case report described daily HUTS without a specified session duration [24].

Despite heterogeneous protocols—ranging from pinpoint peripheral stimulation to whole-body modalities—several common features emerged:

1. Rigorous study designs: Most interventions (RT, PWSTT, DI) employed randomized clinical trials to strengthen evidence quality.
2. Extended training periods: Interventions such as RT and RMT spanned 12 weeks, underscoring the necessity of sustained training for meaningful autonomic adaptation.
3. Protocol diversity: Session lengths varied from seconds (ES, WBC) to weeks (RMT), complicating direct efficacy comparisons but illustrating the breadth of non-invasive approaches.
4. Analogous modalities: ES and AMSS shared similar mechanistic goals of somatosensory activation, while PWSTT and RT both leveraged load-bearing exercise to induce cardiovascular adaptations.

Overall, the wide range of intervention types, timelines, and delivery parameters highlights both the potential and the challenge of standardizing non-invasive rehabilitative strategies for improving BRS in PD.

### 3.4. Study Designs and Protocols

ES was conducted with 16 participants, in which pressure gradually increased on each foot until pain triggered reflex withdrawal. The pressure, measured using a dynamometer, was applied for 6 s at two sites on each foot, repeated four times, totaling approximately 2 min, with an average pressure of 0.58 ± 0.04 kg/mm^2^. A sham protocol followed the same procedure, applying identical pressure and duration to different foot sites, and was also reported as painful by the participants [16]. Similarly, AMSS study employed a similar approach in their study, where patients with moderate-to-severe PD (Hoehn & Yahr stages 2–4) underwent five sessions of AMSS using the Gondola device, a shoe-shaped device with battery-powered motors that apply mechanical pressure to specific areas of the forefoot. During these sessions, cardiovascular autonomic profiles were monitored via electrocardiogram (ECG), beat-to-beat blood pressure, cardiac output, total peripheral resistance, and respiratory activity. Additionally, the Valsalva maneuver and sinus arrhythmia tests were performed, and data were analyzed using spectral and symbolic analysis of HRV and BRS [17].

The potential of WBC as an adjunct to a multidisciplinary rehabilitation program for PD. WBC sessions involved brief exposure to extremely cold temperature (−110 °C) in a cryotherapy chamber for 2 min, integrated with dietary management, physiotherapy, and physical activity, aiming to enhance sympathovagal balance and autonomic function [18]. The effects of repeated short-term DI sessions on autonomic function in PD, with 20 participants undergoing seven 45 min sessions over 25–30 days. The DI procedure involves use of analog microgravity MEDSIM bathtub (MEDSIM system, Institute of BioMedical Problems, Moscow, Russia) with 2 m^3^ of water, covered by a waterproof film. A motor-driven platform initially positioned above the water allows the subject to lie on the film. The platform lowers, immersing the subject in the water while wrapped in the film, with their face and upper thorax floating on the surface during a DI session [19,25]. Blood pressure, heart rate (HR), and ECG readings were recorded, and autonomic function was assessed using HRV analysis, incorporating both linear and nonlinear parameters.

A non-blinded, RCT evaluated the effects of 12 weeks of progressive RT on cardiac autonomic modulation and cardiovascular responses in PD patients. Participants were randomized to either the RT group or a control group and re-evaluated after 12 weeks. Horizontal leg press, squat, rotary calf, lateral pull down, and chest press using isoinertial machines were included in the RT program, with the training load progressively increased from 2 to 4 sets and from 12 to 6 repetitions maximum (RM), to enhance muscle strength. The progression was as follows: weeks 1–2 (2 sets of 12–10 RM), weeks 3–4 (3 sets of 12–10 RM), weeks 5–6 (3 sets of 10–8 RM), weeks 7–10 (4 sets of 10–8 RM), and weeks 11–12 (4 sets of 8–6 RM). Assessments were conducted while patients were in the “on” state of their medication, with post-intervention evaluations for the PDT group performed 48 h after the last training session. Healthy control subjects were assessed once for comparison. The PWSTT consisted of a treadmill with visual biofeedback of step length and an unweighing support system (Biodex Medical System, New York, NY, USA). PWSTT was given during the ‘on’ period of medication. Training sessions were conducted for 30 min per day, 4 days per week, over 4 weeks, and clinical severity was measured using the Unified Parkinson Disease Rating Scale (UPDRS) and gait performance was assessed via the 10 m walk test and treadmill walking [20].

RMT explored to improve pulmonary and cardiovascular autonomic function in PD patients. The initial study focused on strengthening respiratory muscles through specific exercises, while the subsequent study followed the participants for up to 18 months after the cessation of RMT to assess long-term effects [22,23]. HUTS involved the patient gradually increasing the height of the head of the bed over a period of 2 months by 10 cm. Three months after application of HUTS, supine blood pressure and after 3 min of standing was measured. These studies employ a range of interventions, from short-term therapies to long-term training regimens, including physical exercises, mechanical and electrical stimulations, each targeting specific autonomic dysfunctions. The diverse assessment methods, including HRV, blood pressure monitoring, and clinical severity scales, highlight the complex nature of autonomic regulation and the need for personalized therapeutic strategies in clinical practice. Study design and characteristics are summarized in Table 2.

### 3.5. Rehabilitation Effects

ES produced sustained autonomic benefits: it reduced systolic arterial pressure (SAP) and low-frequency SAP during head-up tilt, while augmenting cardiac and vascular sympathetic modulation. These effects persisted for 24 h and were accompanied by significant increases in BRS at rest and during tilt [16].

RT led to a significant reduction in low-frequency respiratory rate after 12 weeks in the PD treatment group, although other HRV and blood pressure measures remained unchanged [21]. Similarly, WBC enhanced autonomic regulation, evidenced by longer R–R intervals and improved frequency-domain indices, without altering blood pressure or plasma catecholamines [18].

PWSTT yielded marked improvements in BRS and motor function (UPDRS scores) following a four-week protocol [20]. DI sessions produced acute reductions in blood pressure and HR, alongside increases in HRV parameters, indicating enhanced autonomic control during immersion [19].

RMT demonstrated correlated gains in pulmonary and cardiovascular autonomic measures: participants exhibited improved BRS post-training, whereas controls showed no change or decline in pulmonary function. However, these BRS gains did not reach statistical significance, and follow-up at 18 months revealed a return toward baseline values [22,23].

AMSS significantly lowered blood pressure and markers of sympathetic activity, while boosting vagal modulation and BRS in PD patients [17]. HUTS improved orthostatic tolerance and nocturnal respiration, though benefits reversed upon discontinuation of the intervention [24].

**Figure 2:**
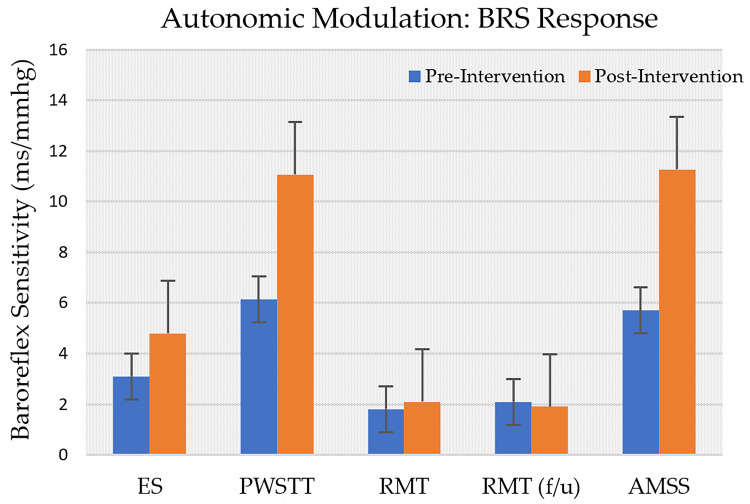
Comparison of Pre-Intervention vs. post-intervention changes in Baroreflex Sensitivity (BRS) through sequence analysis across following interventions: Effective Stimulation (ES), Partial Weight Supported Treadmill Gait Training (PWSTT), and Automated Mechanical Somatosensory Stimulation (AMSS) and through Valsalva Maneuver during Respiratory Muscle Training (RMT) and RMT follow up.

**Figure 3:**
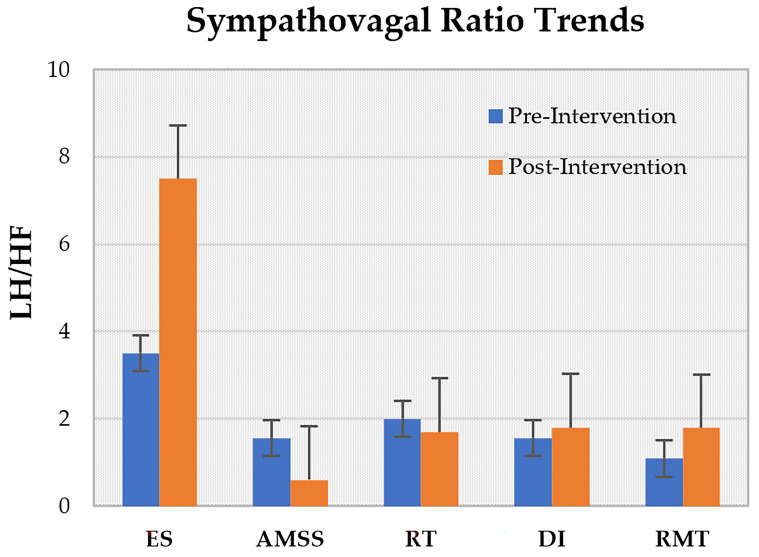
Trends in Pre-Intervention vs. post-intervention ratio between the low frequency and the high frequency components of RR variability (LF/HF) across following interventions: Effective Stimulation (ES), Automated Mechanical Somatosensory Stimulation (AMSS), progressive Resistance Training (RT), repeated short term Dry Immersion (DI), Partial Weight-Supported Treadmill Gait Training (PWSTT), and Respiratory Muscle Training (RMT).

**Figure 4:**
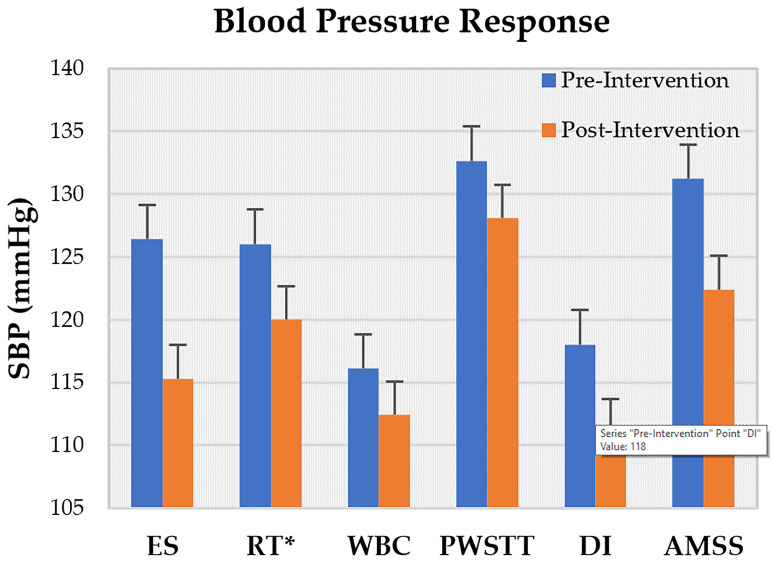
Fall in Systolic Blood Pressure (SBP) changes post-intervention. *RT reported a fall in SBP by 6 ± 3 in the training group without specific measurement values across following interventions: Effective Stimulation (ES), progressive Resistance Training (RT), Whole Body Cryostimulation (WBC), Partial Weight Supported Treadmill Gait Training (PWSTT), repeated short term Dry Immersion (DI) and Automated Mechanical Somatosensory Stimulation (AMSS).

Across studies, ES, PWSTT, and RMT reported post-intervention increases in BRS (Figure 2), with ES showing the most pronounced effect. LF/HF ratios rose after ES, DI, and RMT sessions, but declined following AMSS and RT (Figure 3). On average, SBP decreased by approximately 5% across ES, RT, WBC, PWSTT, DI, and AMSS interventions (Figure 4).

### 3.6. Statistical Analysis

**Figure 5:**
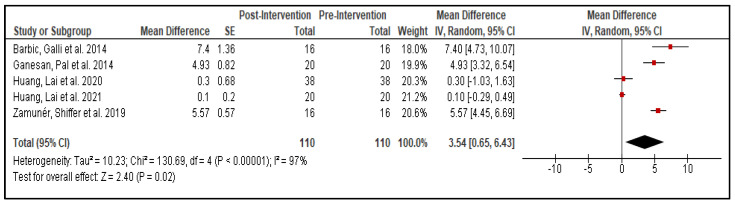
Meta-analysis comparing pre- and post-intervention baroreflex sensitivity (BRS) across studies using the sequence method. The forest plot displays mean differences (MD) with 95% confidence intervals (CI). Positive values indicate improvement in BRS following intervention. Study weights were assigned using the inverse variance method under a random-effects model [16,17,20,22,23].

A random-effects meta-analysis was performed pooling studies that used the BRS sequence method to measure baroreflex sensitivity, regardless of intervention type. Although the included studies employed different interventions, the use of a consistent outcome measure allowed for aggregation. Due to the absence of control or sham groups and cross study designs in the included studies, we performed a pre–post meta-analysis evaluating within-subject changes in BRS. This approach allowed consistent effect size calculation across all eligible studies. The analysis demonstrated a statistically significant improvement in BRS post-intervention (MD = 3.54 ms/mmHg; 95% CI: 0.65 to 6.43; *p* = 0.02), with the overall effect favoring the post-intervention condition. Substantial heterogeneity was observed (I^2^ = 97%), reflecting clinical variability across interventions. All meta-analytic calculations, including effect sizes and heterogeneity estimates, were performed using Review Manager (RevMan) version 5.4.1. The reports of the meta-analysis are summarized in Figure 5.

Common concerns included small sample sizes, lack of blinding, and medication variability. Reporting bias was moderate—few studies had pre-registered protocols or fully reported outcomes/adverse events, limiting confidence. These findings underscore the need for well-powered, transparent, and rigorously controlled trials to validate non-invasive interventions for autonomic dysfunction in PD.

## 4. Discussion

Non-invasive interventions across multiple modalities consistently demonstrated improvements in autonomic regulation and cardiovascular function in PD, providing a safer and more accessible alternative to invasive procedures. These approaches not only enhance patient comfort and adherence but also avoid the risks and side effects associated with pharmacological or surgical treatments. Collectively, the findings support the hypothesis that non-invasive strategies can effectively modulate sympathetic and parasympathetic balance, improving BRS and blood pressure regulation in PD [16,17,18,19,20,21,22,23,24]. Nevertheless, blood pressure dysregulation in PD remains underexplored, and further research is warranted to optimize these rehabilitation methods.

Mechanistic insights emerge when comparing interventions. Both ES and AMSS reduced blood pressure and sympathetic markers while enhancing vagal tone and BRS, with effects persisting 24 h post-stimulation with their mechanisms in line with previous studies [16,17,26,27]. RT and PWSTT similarly reduced orthostatic systolic drops and improved BRS, though RT’s primary impact was on HRV rather than on BRS magnitude [20,21]. WBC increased R–R intervals and improved frequency-domain indices via baroreflex activation through cold-induced sympathetic bursts and compensatory vagal responses [18,28,29]. DI acutely lowered blood pressure and HR increasing HRV, but repeated sessions yielded diminishing returns—suggestive of reduced neuroplasticity in PD [19,30]. This finding is in line with previous studies on game-based rehabilitation [31] and the prior works [25,30,32] on PWSTT for PD. The benefits of RMT in PD patients have been supported by several previous studies [33,34,35], although these studies primarily focused on specific functional areas such as respiratory or cough function, speech function, swallowing, or quality of life [36,37,38]. RMT improved pulmonary and cardiovascular autonomic metrics, though gains in BRS were modest and reverted toward baseline by 18 months [22,23,39]. HUTS enhanced orthostatic tolerance and nocturnal respiration but required ongoing use to maintain benefits [24,40,41].

Underlying physiological mechanisms likely differ by modality. ES and AMSS activate cutaneous and nociceptive afferents projecting to the nucleus tractus solitarii, modulating both sympathetic and parasympathetic outflows [42,43]. PWSTT’s repeated muscle and vascular stretch may enhance vessel elasticity and baroreceptor responsiveness [44], while RT reduces oxidative stress, bolsters mitochondrial function, and increases brain-derived neurotrophic factor, all of which support autonomic stability and motor control. Cold exposure in WBC triggers thermoreceptor-mediated sympathetic activation and subsequent vagal compensation, reinforcing baroreflex loops [28,29]. DI centralizes circulation and lowers peripheral resistance, transiently restoring cardiovascular coupling without exacerbating OH [30,45]. RMT’s effects on autonomic function likely reflect central autonomic network engagement secondary to enhanced respiratory effort and motor cortex activation [46]. Finally, HUTS mitigates orthostatic stress by reducing lower-extremity blood pooling and upper-airway collapse, thereby promoting venous return and stable blood pressure during sleep [40,41,47].

Clinically, these findings suggest that tailored non-invasive protocols could be integrated into PD rehabilitation programs to address autonomic dysfunction. For example, ES and AMSS may benefit patients with both supine hypertension and orthostatic intolerance, potentially reducing reliance on medications. RT and PWSTT can form core exercise prescriptions, improving both cardiovascular regulation and motor function. WBC may serve as a “booster” for autonomic modulation without adverse hemodynamic effects. DI may be applied as an acute intervention for rapid autonomic stabilization, whereas RMT offers combined respiratory and cardiovascular benefits. HUTS presents a low-tech option for improving orthostatic tolerance and nocturnal breathing.

Despite promising results, heterogeneity in protocols, small sample sizes, and short follow-up periods limit generalizability. Future well-powered RCTs should standardize intervention parameters, evaluate dose–response relationships, and assess long-term efficacy and safety. Understanding patient characteristics that predict responsiveness will further enable personalized autonomic rehabilitation in PD.

### 4.1. Limitations

This review highlights several methodological constraints across the included studies:

- ES: Focused on stimulation site specificity without systematically varying stimulus intensity, limiting insight into dose–response relationships.
- RT: Heterogeneity in concurrent medication regimens and absence of long-term follow-up assessments constrain interpretation of sustained autonomic effects.
- WBC: Small cohort size and lack of a control group reduce statistical power and external validity; age-related variability was not explored.
- PWSTT: Participants were not selected based on OH or impaired BRS, limiting applicability to the target dysautonomia population.
- DI: Recruitment challenges and multimorbidity in older subjects precluded an age-matched control group; the individual contributions of immersion versus thermoneutral temperature (32 °C) were not disaggregated.
- RMT: Absence of randomized, blinded, sham-controlled designs and control-group adherence issues in follow-up studies introduce potential bias; baseline differences in age, disease severity, and duration may confound outcomes.
- AMSS: No control or sham-stimulation arm was included, leaving placebo effects unaddressed.
- HUTS: Case-report design and lack of standardized tilt protocols limit generalizability and dose–response characterization.

These limitations underscore the need for larger, randomized, sham-controlled trials with standardized intervention parameters, rigorous follow-up, and well-defined patient selection criteria to validate and generalize the efficacy of non-invasive autonomic rehabilitation in PD.

### 4.2. Future Directions

To advance non-invasive autonomic rehabilitation in PD, future research should focus on the following areas:

1. Standardization and Personalization
  - Develop consensus protocols with clearly defined intervention parameters (e.g., stimulation sites, frequencies, durations).
  - Incorporate individualized titration of stimulation intensity or exercise load based on patient-specific physiological markers (e.g., baseline BRS, hemodynamic responses).
2. Intensive, Multimodal Rehabilitation
  - Design studies that combine complementary interventions (e.g., ES + RT or RMT + HUTS) to target multiple facets of autonomic dysfunction simultaneously.
  - Evaluate “dose–response” effects by comparing standard versus high-intensity regimens to identify optimal training volumes for maximal autonomic adaptation.
3. Mechanistic and Neuromodulatory Investigations
  - Pair clinical trials with mechanistic studies (e.g., neuroimaging, autonomic reflex testing) to uncover neural circuits and molecular pathways responsible for observed benefits.
  - Explore biomarkers (e.g., neurotrophic factors, inflammatory mediators) to monitor treatment response and guide protocol refinement.
4. Long-Term Efficacy and Disease-Modifying Potential
  - Conduct extended follow-up assessments (≥12 months) to determine the durability of autonomic improvements and their impact on clinical outcomes such as fall rates and disease progression.
  - Investigate whether sustained autonomic rehabilitation can modify the trajectory of PD-related cardiovascular decline or delay the onset of disabling symptoms.

By addressing these priorities, the field can move toward evidence-based, patient-centered strategies that reliably restore baroreflex function and improve cardiovascular stability in people living with PD.

## 5. Conclusions

This review demonstrates that a range of non-pharmacological and non-surgical interventions—spanning somatosensory stimulation, exercise modalities, thermal therapies, and positional strategies—can enhance autonomic regulation and motor function in PD. Integrating these therapies into clinical practice holds promise for mitigating orthostatic intolerance and improving patient comfort and compliance. However, the modest gains in BRS observed to date indicate that more intensive, multimodal rehabilitation protocols are likely required to achieve substantial, durable improvements in cardiovascular autonomic control and quality of life for people with PD.

## Figures and Tables

**Table 1:** Participant demographics and PD characteristics.

Author, Year	No. of Participants	Drop Out	Gender (M/F)	Mean Age (Year)	Disease Duration (Year)	BMI (Kg/m^2^)	Hoehn and Yahr Scale
1. Barbic, Galli et al., 2014 [16]	16	NA	8/8	66 ± 3	13 ± 1	23 ± 1	2–3
2. Kanegusuku, Silva-Batista et al., 2017 [21]	30	3	22/5	65 ± 8	8.7 ± 4.7	25.6 ± 3.6	2–3
3. Piterà, Cremascoli et al., 2024 [18]	17	4	6/7	64.5 ± 9	5.4 ± 7.3	26.18 ± 3.91	1–3
4. Ganesan, Pal et al., 2014 [20]	60	NA	46/14	58.15 ± 8.7	5.3 ± 3.4	23.57 ± 3.92	1–3
5. Gerasimova-Meigal, Meigal et al., 2021 [19]	20	NA	13/7	61 ± 6	4.5 ± 1.1	27.42 ± 3.7	1–3
6. Huang, Lai et al., 2020 [22]	75	NA	29/33	64.1 ± 9.9	5.4 ± 4.4	24.2 ± 4.35	2–3
7. Huang, Lai et al., 2021 [23]	75	23	23/29	64.9 ± 9.85	5.25 ±	NA	2–3
8. Zamunér, Shiffer et al., 2019 [17]	23	7	6/10	66.2 ± 9.4	7 ± 3.5	24.2 ± 2.8	2–4
9. van der Stam, Shmuely et al., 2024 [24]	1	NA	M	69.00	10	NA	2

NA = Not Applicable.

**Table 2:** Study design, intervention delivered and their delivery protocols.

Author, Year	Study Design	Intervention	Length of Delivery	No. and Frequency of Sessions	Each Session Time	Quality Rating *
1. Barbic, Galli et al., 2014 [16]	Randomized Clinical Trial	ES	2 min	4; 4	6 s	Moderate
2. Kanegusuku, Silva-Batista et al., 2017 [21]	Randomized Clinical trial	RT	12 weeks	24; 2 days/week	NA	High
3. Piterà, Cremascoli et al., 2024 [18]	Pilot Study	WBC	1 week	10; 2/day	2 min	Moderate
4. Ganesan, Pal et al., 2014 [20]	Randomized Clinical Trial	PWSTT	4 weeks	16; 4 days/week	30 min	High
5. Gerasimova-Meigal, Meigal et al., 2021 [19]	Controlled Clinical Trial	DI	4 weeks	7; 2/week	45 min	Moderate
6. Huang, Lai et al., 2020 [22]	Prospective Case–Control study	RMT	12 weeks	120; 2/day	30 min	Moderate
7. Huang, Lai et al., 2021 [23]	Prospective Case–Control study	RMT	12 weeks	120; 2/day	30 min	Moderate
8. Zamunér, Shiffer et al., 2019 [17]	Interventional Model	AMSS	12 days	5; 2/week	NA	Moderate
9. van der Stam, Shmuely et al., 2024 [24]	Case Report	HUTS	∞	∞; 1/day	NA	N/A

* Quality of the studies was assessed using the Downs and Black checklist for clinical trials, based on the total score (maximum 27), studies were categorized as follows: high quality (21–27), moderate quality (15–20), and low quality (0–14). NA = Not Applicable.

## Data Availability

Extracted data and materials used for generation of results is available from the corresponding author upon request. The protocol for the review was not prepared.

## Abbreviations

The following abbreviations are used in this manuscript:

PD	Parkinson’s Disease
OH	Orthostatic Hypotension
SBP	Systolic Blood Pressure
DBP	Diastolic Blood Pressure
BRS	Baroreflex Sensitivity
UPDRS	Unified Parkinson Disease Rating Scale
ES	Effective Stimulation
RT	progressive Resistant Training
WBC	Whole Body Cryostimulation
PWSTT	Partial Weight Supported Treadmill Training
DI	Dry Immersion
RMT	Respiratory Motor Training
HUTS	Head Up Tilt Sleeping
AMSS	Automated Mechanical Somatosensory Stimulation
RCT	Randomized Clinical Trial
ECG	Electrocardiograph
SAP	Systolic Arterial Pressure
HRV	Heart Rate Variability
HR	Heart Rate
LF	Low Frequency
HF	High Frequency
